# Social and anthropometric factors explaining racial/ethnical differences in birth weight in the United States

**DOI:** 10.1038/srep46657

**Published:** 2017-04-21

**Authors:** Naho Morisaki, Ichiro Kawachi, Emily Oken, Takeo Fujiwara

**Affiliations:** 1 National Center for Child Health and Development, Tokyo, Japan; 2 Harvard School of Public Health, Boston, Massachusetts, USA; 3 Harvard Medical School and Harvard Pilgrim Health Care Institute, Boston, Massachusetts, USA

## Abstract

Though disparities in birth weight by race/ethnicity have been extensively reported in the United States, few studies have systematically investigated factors attributing to its variability. For 10,638,415 singleton infants born during 2009–2012 in the United States, we examined birth weight differences among 14 races and ethnicities (non-Hispanic white, non-Hispanic Black, American Indian, Asian Indian, Chinese, Filipino, Japanese, Korean, Vietnamese, Hawaiian, Guamanian, Mexican, Puerto Rican and Cuban), after sequentially adjusting for maternal, socio-economic and behavioral factors. Average birthweight of non-Hispanic white infants was 3381 g, while for other races/ethnicities birth weight ranged from being 289 g smaller in Japanese to 126 g larger in Samoan infants. Factors explaining differences of more than 50 grams in birth weight compared to white infants were: gestational age for black infants, height and body mass index for all Asian and Samoan mothers, and gestational weight gain for Japanese mothers. Difference in maternal age, parity, socioeconomic and behavioral characteristics did not account for significant portion of birthweight variations for any race. Our findings suggest that differences in maternal anthropometrics, gestational weight gain, and preterm birth rate, but not in maternal age, parity, socioeconomic or behavioral characteristics contribute to racial/ethnic differences in birthweight.

Disparities in birthweight outcomes by race and ethnicity have been extensively reported^1^,^2^,^3^,^4^,^5^. However, recent studies have found that birth weight variability among nations was relatively small when limited to low risk women^6^,^7^.

The United States (US) is one of the most racially and ethnically diverse populations, with over 3 million infants of various races/ethnicities and national origins born each year. Numerous studies have found interracial differences in birth weight. For example, black and Asian infants are generally smaller than white infants^1^,^2^,^8^, and heterogeneity has been shown within the Asian^9^,^10^,^11^ and Hispanic subgroups as well^12^,^13^. However, few studies have quantitatively assessed the contribution of different parental characteristics to account for birth weight variability. Prior studies in multi-ethnic populations such as the California Bay Area^1^,^12^,^14^,^15^,^16^,^17^ and New York^18^, or studies using the US National Natality File^9^,^13^,^19^ have only looked at broadly aggregated race/ethnic groups such as “Asian” and “Hispanics”, or were not able to consider the contribution of maternal anthropometrics and gestational weight gain, which had not been recorded in the previous birth certificate.

Recently the US national birth certificate database (National Natality File^20^) which records detailed characteristics of all births in the US, has begun providing information on maternal height and pre-pregnancy weight. As maternal anthropometrics as well as socioeconomic and behavioral factors are strong predictors of birth weight, and as these factors vary by race/ethnicity as well, we sought to use these data to quantify how maternal anthropometric, socioeconomic, and behavioral factors contribute to racial variability in birth weight in the US.

## Results

The total number of singleton births with data available was 10,638,415. Of these, 67% of infants were classified as having parents with a matching distinctive race; 42% were non-Hispanic white, 10% were Mexican, 7.5% were non-Hispanic black, and the proportion of all other races/ethnicities ranged from 0.002% (Hawaiian) to 1.3% (Indian). 15% had mixed race (parents were of different race or at least one parent was of mixed race), and 20% had at least one parent with race unknown.

Tables 1 and 2 show selected maternal and infant characteristics by race/ethnicity. Wide variation was seen in socioeconomic, behavioral and obstetric characteristics. For maternal characteristics, American Indian mothers were youngest (mean 25.4 years) and Japanese were oldest (mean 34.0 years); 14.9% of American Indian mothers smoked, compared to only 0.1% in Asian Indians and Chinese; and 66.9% of American Indian mothers did not have any college education, compared to 84.0% of Korean mothers having a college degree or above. In addition, 66.9% of American Indian mothers were not married compared to 1.7% among Asian Indians; 84.4% of Japanese initiated prenatal care during the 1^st^ trimester while 3.1% of Guamanians received no prenatal care; 2.3% of white women delivered outside of hospital compared to 0.1% of Samoans, and 66.1% of American Indians were Medicaid users compared to only 6.0% of Japanese.

Samoans had the largest proportion of overweight (BMI > 25 kg/m^2^) and obese (BMI > 30 kg/m^2^) mothers (89.0%) and Japanese had the largest number of underweight (BMI < 18.5 kg/m^2^) (19.4%) mothers. Samoans (167.5 cm) were tallest and Vietnamese shortest (156.7 cm). Koreans (47.9%) had the largest and Samoans (25.8%) the smallest proportion of null para mothers.

For obstetric characteristics, gestational weight gain was highest among Samoans (mean 15.6 kg) and lowest among Japanese (mean 11.2 kg). Pre-pregnancy and gestational diabetes was most frequent in Samoans (9.2%) and lowest in Japanese (3.2%). Pre-pregnancy and gestational hypertension was most frequent in black mothers (8.0%) and lowest in Japanese (1.2%) mothers.

Mean birth weight for non-hispanic white infants was 3381 g, and ranged from 3093 g in Japanese to 3507 g in Samoans. Only Hawaiians had more female infants than male infants. The preterm delivery rate was highest in black infants (14.2%) and lowest in Koreans (5.4%).

**Figure 1:**
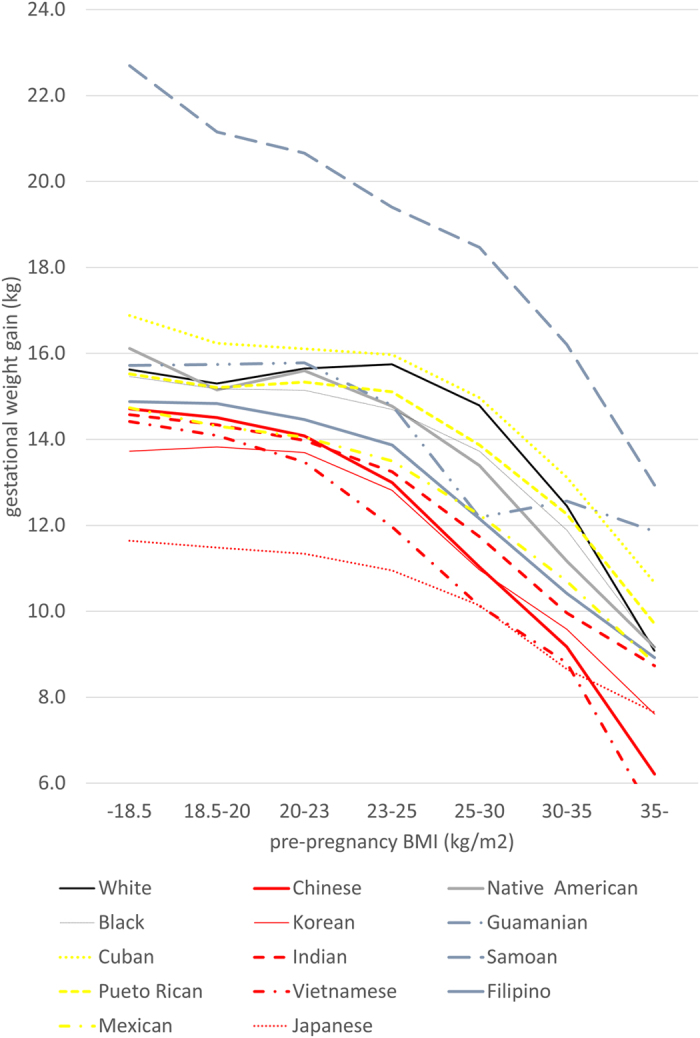
Average Gestational Weight Gain by BMI among 14 races. Analysis of Singleton Births in the United States, 2009–2012.

As distribution of pre-pregnancy BMI differed by race/ethnicity, and gestational weight gain recommendations differ by BMI, we have shown the distribution of average gestational weight gain stratified by BMI and race/ethnicity in Fig. 1. Hawaiians are not shown due to small sample size. Average gestational weight gain declined as BMI increased. The largest average gestational weight gain was found among Samoans in all BMI categories, and the smallest average gestational weight gain was overweight Japanese and Vietnamese. Average gestational weight gain was 11.6–16.9 kg for underweight, 11.3–16.1 kg for normal weight, and 9.8–14.9 kg for overweight and obese mothers, except for the extreme outlier, Samoans.

**Figure 2:**
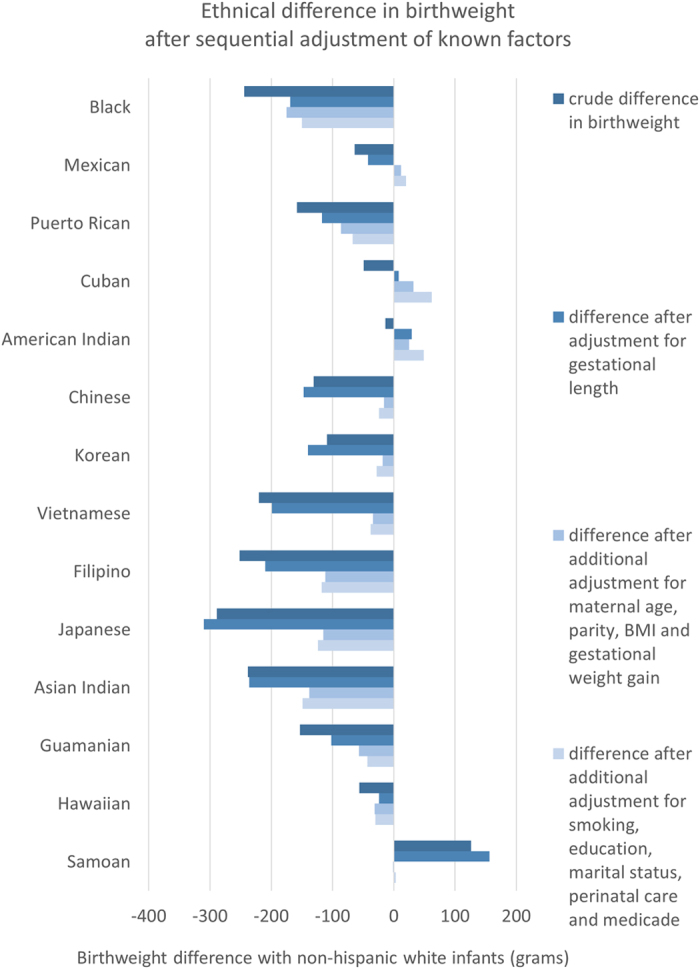
Racial differences in birth weight after sequential adjustment of gestational age, maternal age, parity, BMI, gestational weight gain, and socioeconomic and behavioral characteristics. Analysis of Singleton Births in the United States, 2009–2012.

Next, in Fig. 2 we display the average birth weight for the 14 included races/ethnic groups, before and after adjustment for maternal, obstetric, societal and behavioral factors (numbers are shown in Appendix 1). The average birth weight differed from non-hispanic white infants by over 100 g for all races/ethnicities other than Koreans, Mexicans, Cubans and American Indians. Japanese were smallest being 289 g smaller on average, and only Samoans had an average birth weight larger (+126 g) than white infants. For races/ethnicities other than Puerto Ricans, this difference persisted after adjustment for gestational age, and after additional adjustment for maternal age and parity. However, after additional adjustment by maternal pre-pregnancy size, the difference in average birth weight by over 100 g remained only in Japanese, Asian Indian, Filipino and black infants, with black infants being smallest (168 g smaller than white infants) and Cubans being largest (44 g larger than white infants). After adjustment for all factors, average birth weight difference between the largest and smallest race/ethnicity decreased from 415 g to 213 g. Nearly 40% of birthweight variability could be explained by these factors (R^2^ = 0.38 in final model), and nearly 60% of the variability in birthweight due to race/ethnicity could also be explained by these factors (partial R^2^ of race decreased from 0.025 in crude model to 0.010 in final model).

We also compared average birth weight z-scores limited to gestational weeks 34–41 across the 14 races/ethnicities (shown in Appendix 2). Average birth weight z-scores differed from white infants by over 0.1 SD in all races/ethnicities other than Mexicans, Cubans, and American Indians. Japanese were smallest (−0.69 SD), and Samoans (+0.31 SD) and American Indians (+0.02 SD) were larger than white infants. These differences also persisted after adjustment for gestational age, and after additional adjustment for maternal age and parity. After additional adjustment by height and BMI, difference in birth weight z-scores over 0.1 SD remained only in Japanese, Asian Indian, Filipino and black infants, with black infants being smallest (−0.37 SD smaller than white infants) and American Indians being largest (0.08 SD larger than white infants). For black infants, adjustment by all factors barely changed the birth weight discrepancy with white infants.

Regarding characteristics that correlated with racial difference in average birth weight by over 50 g, we noted the following: shorter gestational age for black infants [+81 g], lower maternal BMI and height for Asian infants (Vietnamese [+171 g], Japanese [+150 g], Chinese [+118 g], Filipino [+107 g], Koreans [+102 g], and Asian Indian [+70 g]), higher maternal BMI & height for Pacific Islanders (Guamanian [+68 g] and Samoans [−99 g]); and lower gestational weight gain for the Japanese (+60 g). Distribution of maternal age, parity and other socioeconomic and behavioral factors (smoking, education, marital status, initiation of antenatal care, and medical insurance status) barely added to explaining any difference in average birth weight for any race/ethnic group.

## Discussion

To our knowledge, this is the first comprehensive study using a US population sample to explore parental characteristics associated with race/ethnic variations in birth weight. Background maternal characteristics including anthropometric, obstetric, socioeconomic, and behavioral factors varied widely. Factors that explained over 50 g of the difference in mean birth weight compared to whites included: gestational age for black infants, maternal height and BMI for all Asian and Samoan mothers, and gestational weight gain for Japanese mothers. Nevertheless, socioeconomic and behavioral factors failed to account for substantial (i.e. >50 g) differences in birthweight between race/ethnic groups.

Our study highlights potential target areas for maternal interventions to reduce adverse birth outcomes. For example, gestational weight gain was markedly higher in Samoan women. Adult Samoans have a higher prevalence of overweight/obesity compared to other race/ethnic groups^9^,^19^, with higher birth weight being a risk factor for obesity^21^. Japanese women had low gestational weight gain, especially among underweight women, and this is most likely a significant contributing factor to the small size of Japanese infants, which is smallest even among Asian infants. Appropriate gestational weight gain guidance may reduce the number of infants that are too large or too small. High pre-pregnancy BMI was prominent in Pacific Islander, American Indian, white and black mothers. These races/ethnic groups also are also affected by rising rates of obesity, and may benefit from nutritional guidance targeting women of child-bearing age. As ethnicity is closely related to culture, including eating habits as well as norms about appearance^22^,^23^,^24^, acknowledging how ethnicity may be related to maternal nutritional status before as well as during pregnancy, may help improve birth outcomes for all ethnicities.

The preterm birth rate was highest among black mothers, which is consistent with previous reports^1^,^8^. However, even after taking into account differences in gestational age, black infants were substantially smaller than non-Hispanic infants, and after adjusting for all social and anthropometric factors captured in the birth certificate, they ended up as smallest among all races. Although our study failed to identify the reasons for this disparity, other researchers have suggested that cumulative socioeconomic disadvantage as well as the legacy of racial discrimination may be contributory factors^8^,^16^.

A study in the United Kingdom used a similar approach to examine the contribution of socioeconomic, maternal anthropometric and behavioral factors to race/ethnic differences in mean birth weight^4^. It found that socioeconomic factors were important for black infants, and maternal height and weight were important for Asian Indian infants^4^. However, this study included a small sample size (16,157 births), and fewer ethnic groups. In the Netherlands, the difference between Dutch and race/ethnic minorities was reduced from over 400 g to under 200 g after taking into account maternal and paternal height, BMI, gestational age, as well as education level, maternal smoking, maternal alcohol use, marital status, maternal age and parity^25^. Distribution of maternal height was a prominent factor for all minor races/ethnicities, explaining over a 100 g difference in average birth weight compared to white infants^26^.

Although the specific minor races/ethnic groups within these two countries differ from the United States and are not directly comparable, both studies supported our findings that racial differences in maternal anthropometrics may be the largest driver in racial variability in fetal growth, and consideration of maternal size reduces the disparity in birth weight distribution across races/ethnicities.

Currently a unified national birth weight reference is used to diagnose an infant as too small or too large for gestational age, both which are risks for unfavorable consequences^27^,^28^,^29^,^30^,^31^,^32^,^33^,^34^,^35^,^36^. There is criticism this definition may be misleading in a multi-ethnic population, as being small or large at birth may be merely due to the mother being small or large, rather than due to any pathological factor^2^. While recently a “race-specific” birthweight reference has been proposed^37^, we have shown differences in birth weight is more complex than the broad categories of race/ethnicity (non-Hispanic white, black, Hispanic, Asian) used in this new reference. Also, in countries with large number of ethnicities of the residents as well as frequent interracial marriage, usage of an “ethnically customized” birthweight reference is unrealistic. Therefore, we propose that any customization of the birth weight reference should be according to maternal and possibly also paternal anthropometrics, which may help in reducing this mismatch.

We also found that the magnitude of explanation of birth weight variability by maternal factors differed by race/ethnicity. Of the 11 races/ethnic groups with over a 100 g difference in mean birth weight compared to white infants, our model explained over 80% of the difference for Vietnamese, Chinese, and Samoans, while over 60% of the difference remained unexplained for black and Asian Indian infants. Many previous studies failed to explain the birth weight difference between white and black infants, even after adjustment for maternal, social and behavioral factors^1^,^8^,^38^. Lower birth weight of Asian Indian infants compared to white, as well as other Asian infants, has been widely documented in the US and elsewhere^4^,^9^,^10^,^11^,^19^,^39^,^40^. One study in the United Kingdom showed maternal and infant factors were important in explaining some of the birth weight difference between Asian Indian and white infants^4^, however the unexplained difference (142 g) was very similar to that in our study (149 g). Additional studies with more detailed measures may be needed to explain these persistent differences.

Our study has several limitations. First, we did not have information on paternal anthropometrics. As we found maternal anthropometrics affect mean birth weight differences among races/ethnicities, paternal anthropometrics may be one of the factors causing the unexplained disparity among races/ethnic groups. Second, although we found that maternal anthropometrics, gestational weight gain and gestational length explained a substantial portion of racial differences in birth weight, as we were not able to differentiate US-born infants and non-US births, we do not know whether these characteristic differences are genetic, or due to cultural or environmental diversity. Third, although our study was based on national data, our database was limited to states that used the 2003 revision of the US birth certificate, hence our coverage was 66–86% of all births. Finally, the natality data is a combination of forms filled in by the mother as well as by staff of the birth facility, with no strict standardization of completeness or accuracy, and differences in completeness and accuracy between races may have caused bias. Increased standardization on by whom and how thoroughly these forms should be filled out, would improve accuracy in research results derived from natality data in future studies.

Despite these limitations, this is the first and largest study to look at birth weight among races/ethnicities that investigates how maternal characteristics including anthropometrics explain variability in birth weight in the United States. Further study is needed to reveal whether such differences are inherited and thus not modifiable, or whether the unexplained racial differences reflect exposures that were not captured in the birth records, e.g. cumulative exposure to socioeconomic disadvantage across the life course, as well as the historical and ongoing legacy of race/ethnic discrimination of minority groups in society.

## Methods

For this study we used the National Natality File^20^, a publically available database of US birth certificates available from the Vital Statistics branch at the Centers for Disease Control and Prevention. We used data from all singleton live births 2009–2012.

Data was restricted to those births recorded on the 2003 revised birth certificates. This restriction was applied because the reporting of multiple-race/ethnicity categories and details on maternal anthropometrics, such as maternal height, pre-pregnancy weight, delivery weight, are included in the 2003 revised version of the birth certificate but not the older 1989 version. In 2009, 28 of 50 states in the US were using the 2003 revised version of the birth certificate, and this increased to 38 plus Washington DC in 2012, representing 66% of all US births in 2009 and 86% in 2012.

Of 12,375,027 singleton births within 2009–2012, the following subjects were excluded from analysis: missing information on birthweight (n = 13,154), missing information on gestational age (n = 13,196), delivered before 22 weeks or after 42 weeks (n = 696,536), missing information on mother’s height or body mass index (BMI) (n = 536,238), missing information on gestational weight gain (n = 178,809), missing information on birth facility or initiation of prenatal care (n = 298,679). Thus, analysis was done on the remaining 10,638,415 infants.

The 2003 US birth certificate documents self-reported maternal and paternal race/ethnicity, and Hispanic origin. Race is recorded as either single or ‘bridged’ race/ethnicity, with ‘bridged’ meaning mixed race/ethnicity. Single racial or ethnic categories included white, black or African American, American Indian/Alaskan Native, Asian Indian, Chinese, Filipino, Japanese, Korean, Vietnamese, Native Hawaiian, Guamanian, Other Asian, or Other Pacific Islander. Hispanic origin is documented as Hispanic (Mexican, Puerto Rican, Cuban, Central or South American, Other and Unknown Hispanic, Origin unknown) or non-Hispanic.

Using these variables, we classified both maternal and paternal race/ethnicity as follows: non-Hispanic white, non-Hispanic black, Mexican, Puerto Rican, Cuban, American Indian, Asian Indian, Chinese, Filipino, Guamanian, Japanese, Korean, Vietnamese, native Hawaiian. Other race/ethnic groups (such as “Other Asian” “South American”) or unknown race/ethnicity, non-white Hispanics, and bridged race/ethnicity were classified as “Mixed, Other or Unknown Race”.

As our interest was mainly in infants of parents with a distinct race/ethnicity, we defined “infant race” as infants born to parents with a matching single race/ethnicity, or who were assigned their parents’ race/ethnicity. All other infants who had either at least one parent with “Mixed, Other or Unknown Race”, or parents of different race/ethnicities, were classified as “Mixed, Other or Unknown Race”.

The following socioeconomic and behavioral factors were used: education (high school or below, some college education, college degree or higher, unknown), marital status (married, single or divorced, unknown), smoking status (yes, no, unknown), medical insurance status (Medicaid user, private insurance, self-pay, unknown), and initiation of prenatal care (1^st^ trimester, 2^nd^ trimester, 3^rd^ trimester, none, unknown). Obstetric characteristics were also categorized when used in the multivariate analysis models: birth order (1^st^, 2^nd^ or above), maternal age (in years), BMI (kg/m^2^, in integral units), height (in cm), gestational age (in weeks) and gestational weight gain (in kgs).

Birth weight z-scores were calculated using the US birth weight reference by Oken, which is based on natal data from births 1999–2000. Multivariate linear regression was used to calculate the association of race/ethnicity with birth weight and birth weight z-scores, after adjusting for potential confounders. Further, partial R^2^ derived from multivariate linear regression models including and excluding race/ethnicity was used to characterize the racial variability in birth weight after adjusting for potential confounders. For these models, we selected non-Hispanic white race/ethnicity a priori as the reference group due to its large population. Potential confounders adjusted for included maternal characteristics (age, height, pre-pregnancy BMI, parity, gestational weight gain), and socioeconomic and behavioral factors (education, marital status, Medicaid use, smoking, initiation of prenatal care). We did not use gestational hypertension and gestational diabetes because we considered them to be mediating factors rather than confounders.

The protocol for this study was developed according to “Ethical Guidelines for Medical and Health Research Involving Human Subjects” and approved by the Institutional Review Board at the National Center for Child Health and Development, Tokyo, Japan (Project #2014-09). All descriptive and statistical analyses were performed using STATA version 13 (STATA Corp, College Station, TX). Statistical significance was set at the 0.05 level, and all statistical tests were two-tailed.

## Additional Information

**How to cite this article:** Morisaki, N. *et al*. Social and anthropometric factors explaining racial/ethnical differences in birth weight in the United States. *Sci. Rep.* **7**, 46657; doi: 10.1038/srep46657 (2017).

**Publisher's note:** Springer Nature remains neutral with regard to jurisdictional claims in published maps and institutional affiliations.

## Supplementary Material

Appendix file

## Figures and Tables

**Table 1:** Maternal, Societal, Behavioral and Obstetric Characteristics by Selected Race (White, Black, Mexican, Puerto Rican, Cuban, American Indian, Hawaiian, Guamanian and Samoan).

N	Maternal and paternal race								
	White	Black	Mexican	Puerto Rican	Cuban	Native American	Hawaiian	Guamanian	Samoan
	4,507,154	797,933	1,034,662	26,969	20,619	29,315	152	904	2,481
Maternal characteristics									
Age (yrs) [mean (SD)]	29 (6)	27 (6)	27 (6)	27 (6)	29 (6)	25 (6)	28 (6)	27 (6)	28 (6)
BMI (kg/m^2^) [mean (SD)]	26 (6)	28 (7)	27 (6)	27 (6)	25 (5)	28 (7)	28 (6)	27 (6)	33 (7)
Height (cm) [mean (SD)]	165 (7)	164 (7)	159 (7)	161 (7)	162 (6)	163 (7)	162 (7)	157 (7)	168 (7)
Number of previous deliveries									
0 (%)	42	37	32	41	47	32	30	27	26
1 (%)	33	29	30	31	37	26	29	29	23
2<= (%)	25	34	39	28	16	42	41	45	51
Socioeconomic and behavioral characteristics									
Smoking (%)	10.9	5.6	1.0	5.7	2.0	14.9	4.9	7.7	6.5
Not married (%)	21	60	45	50	42	67	40	45	26
Maternal education									
College degree (%)	51	26	13	30	40	13	22	16	16
College credit (%)	21	28	16	25	18	24	21	19	27
High school (%)	28	46	71	46	42	62	58	65	58
Medicaid user (%)	27	58	61	51	50	66	44	37	51
Month of initiation of prenatal care									
1–3 months (%)	81	67	71	76	85	56	65	58	51
4–6 months (%)	16	25	23	20	13	32	28	30	33
7–9 months (%)	3	6	5	4	2	10	6	8	14
None (%)	0.7	2.1	1.8	0.7	0.4	1.9	1.6	3.1	2.0
Obstetric characteristics									
Birth weight (grams) [mean]	3381	3136	3317	3223	3331	3367	3325	3228	3507
Male infant (%)	51	51	51	52	51	51	48	52	54
Preterm delivery (%)	8	14	10	11	12	13	9	13	12
Gestational weight gain (kg) [mean]	14.4	12.9	12.5	13.9	15.1	12.8	13.4	13.1	15.6

Analysis of Singleton Births in the United States, 2009–2012. BMI: body mass index.

**Table 2:** Maternal, Societal, Behavioral and Obstetric Characteristics by Race Among Asian and Mixed-Race Infants, and Infants of Other or Unknown Race.

N	Maternal and paternal race						
	Indian	Chinese	Korean	Japanese	Vietnamese	Filipino	Mixed, other or unknown race
	135,502	93,805	27,219	6,171	43,437	43,317	3,868,775
Maternal characteristics							
Age (yrs) [mean (SD)]	30 (4)	32 (5)	33 (4)	34 (4)	32 (5)	32 (5)	26 (6)
BMI(kg/m^2^)[mean(SD)]	24 (4)	21 (3)	22 (3)	21 (3)	21 (3)	24 (4)	27 (7)
Height(cm)[mean (SD)]	161 (6)	161 (6)	162 (5)	159 (6)	157 (6)	158 (6)	162 (7)
Number of previous deliveries							
0 (%)	50	49	48	49	42	39	37
1 (%)	40	40	37	38	28	36	33
2<= (%)	11	11	15	13	30	25	31
Socioeconomic and behavioral characteristics							
Smoking (%)	0.1	0.1	0.4	0.4	0.2	0.6	11.5
Not married (%)	1.7	9.1	2.6	2.1	12	14	65
Maternal education							
College degree (%)	81	65	84	80	41	71	21
College credit (%)	6	7	9	10	15	19	21
High school (%)	13	28	7	9	44	11	58
Medicaid user (%)	17	33	21	6	37	18	58
Month of initiation of prenatal care							
1–3 months (%)	81	80	81	84	77	82	65
4–6 months (%)	15	16	14	12	20	15	26
7–9 months (%)	3.7	3.7	4.5	3.1	2.9	2.8	6.8
None (%)	0.8	0.4	0.6	0.2	0.9	0.6	2.8
Obstetric characteristics							
Birthweight (g) [mean]	3143	3250	3272	3093	3161	3129	3246
Male infant (%)	52	52	52	51	52	52	51
Preterm delivery (%)	8	6	5	6	9	11	12
Gestational weight gain (kg) [mean]	12.9	13.7	13.2	11.2	13.1	13.3	13.3

Analysis of Singleton Births in the United States, 2009–2012. BMI: body mass index.
