# Supplementary material for: Social and anthropometric factors explaining racial/ethnical differences in birth weight in the United States
Source: Sci Rep. 2017 Apr 21;7:46657. doi: 10.1038/srep46657 (PMC5399358; doi:10.1038/srep46657)
Supplement: Appendix file [file srep46657-s1.pdf]

Appendix 1: Average Birth Weight Difference in Grams of 14 Races Compared to White Infants. Analysis of Singleton Births in the United States, 2009–2012.

	Crude Estimate	Additionally adjusted for gestational age	Additionally adjusted for maternal age and parity	Additionally adjusted for height and BMI	Additionally adjusted for gestational weight gain	Additionally adjusted for socio-economic and behavioral factors
Japanese	-289	-310	-313	-173	-115	-124
95% CI	[-302 - -275]**	[-321 - -298]**	[-325 - -302]**	[-184 - -162]**	[-126 - -104]**	[-135 - -114]**
Filipino	-252	-210	-219	-122	-112	-118
95% CI	[-257 - -247]**	[-214 - -206]**	[-223 - -214]**	[-126 - -117]**	[-116 - -108]**	[-122 - -114]**
Black	-244	-169	-168	-183	-175	-150
95% CI	[-246 - -243]**	[-170 - -168]**	[-170 - -167]**	[-184 - -182]**	[-176 - -174]**	[-151 - -149]**
Asian Indian	-238	-236	-228	-165	-138	-149
95% CI	[-241 - -235]**	[-238 - -233]**	[-230 - -225]**	[-167 - -162]**	[-141 - -136]**	[-151 - -147]**
Vietnamese	-220	-199	-208	-52	-34	-38
95% CI	[-225 - -215]**	[-204 - -195]**	[-213 - -204]**	[-56 - -47]**	[-38 - -30]**	[-42 - -34]**
Guamanian	-153	-102	-117	-53	-57	-43
95% CI	[-189 - -118]**	[-133 - -72]**	[-146 - -87]**	[-82 - -24]**	[-86 - -29]**	[-71 - -14]**
Chinese	-131	-147	-146	-35	-16	-24
95% CI	[-134 - -127]**	[-150 - -144]**	[-149 - -143]**	[-38 - -32]**	[-19 - -13]**	[-26 - -21]**
Hawaiian	-56	-24	-34	-32	-31	-30
95% CI	[-142 - 31]	[-98 - 49]	[-106 - 38]	[-103 - 38]	[-101 - 38]	[-99 - 39]
Puerto Rican	-158	-117	-116	-86	-86	-67
95% CI	[-165 - -152]**	[-123 - -112]**	[-122 - -111]**	[-91 - -80]**	[-91 - -81]**	[-72 - -62]**
Korean	-109	-140	-142	-46	-18	-28
95% CI	[-115 - -102]**	[-146 - -135]**	[-148 - -137]**	[-51 - -41]**	[-23 - -13]**	[-33 - -22]**
Mexican	-64	-42	-49	-5	12	20
95% CI	[-65 - -63]**	[-43 - -41]**	[-50 - -48]**	[-6 - -4]**	[11 - 13]**	[19 - 21]**
Cuban	-49	8	15	44	32	62
95% CI	[-57 - -42]**	[2 - 15]**	[9 - 21]**	[38 - 51]**	[26 - 38]**	[57 - 68]**
American Indian	-14	29	26	17	25	49
95% CI	[-20 - -8]**	[24 - 35]**	[20 - 31]**	[11 - 22]**	[20 - 30]**	[44 - 54]**
Samoan	126	156	136	42	-1	3
95% CI	[104 - 147]**	[138 - 174]**	[118 - 154]**	[24 - 59]**	[-18 - 16]	[-14 - 20]

BMI: body mass index, 95% CI: 95% confidence interval * $P < 0.05$; ** $P < 0.01$

Appendix 2. Average Birth Weight Z-Score Difference of 14 Races Compared to White Infants. Analysis of Singleton Births in the United States, 2009–2012.

	Crude Estimate	Additionally adjusted for maternal age and parity	Additionally adjusted for height and BMI	Additionally adjusted for gestational weight gain	Additionally adjusted for socio-economic and behavioral factors
Japanese	-0.69	-0.70	-0.37	-0.24	-0.29
95% CI	[-0.71 - -0.66]**	[-0.72 - -0.68]**	[-0.39 - -0.34]**	[-0.26 - -0.22]**	[-0.31 - -0.27]**
Asian Indian	-0.52	-0.50	-0.35	-0.29	-0.34
95% CI	[-0.52 - -0.51]**	[-0.51 - -0.50]**	[-0.35 - -0.34]**	[-0.29 - -0.28]**	[-0.34 - -0.33]**
Filipino	-0.45	-0.47	-0.23	-0.20	-0.24
95% CI	[-0.45 - -0.44]**	[-0.48 - -0.46]**	[-0.24 - -0.22]**	[-0.21 - -0.19]**	[-0.25 - -0.24]**
Vietnamese	-0.44	-0.47	-0.09	-0.04	-0.08
95% CI	[-0.45 - -0.43]**	[-0.47 - -0.46]**	[-0.10 - -0.08]**	[-0.05 - -0.03]**	[-0.09 - -0.07]**
Black	-0.37	-0.37	-0.40	-0.38	-0.37
95% CI	[-0.37 - -0.37]**	[-0.37 - -0.37]**	[-0.40 - -0.40]**	[-0.38 - -0.38]**	[-0.37 - -0.36]**
Chinese	-0.32	-0.32	-0.06	-0.02	-0.05
95% CI	[-0.33 - -0.32]**	[-0.33 - -0.31]**	[-0.06 - -0.05]**	[-0.02 - -0.01]**	[-0.06 - -0.04]**
Korean	-0.31	-0.32	-0.09	-0.03	-0.07
95% CI	[-0.32 - -0.30]**	[-0.33 - -0.30]**	[-0.10 - -0.08]**	[-0.04 - -0.02]**	[-0.08 - -0.06]**
Guamanian	-0.24	-0.27	-0.11	-0.10	-0.10
95% CI	[-0.29 - -0.19]**	[-0.32 - -0.21]**	[-0.16 - -0.05]**	[-0.15 - -0.05]**	[-0.16 - -0.05]**
Puerto Rican	-0.19	-0.19	-0.11	-0.12	-0.12
95% CI	[-0.20 - -0.19]**	[-0.19 - -0.18]**	[-0.12 - -0.11]**	[-0.13 - -0.11]**	[-0.13 - -0.11]**
Hawaiian	-0.13	-0.15	-0.12	-0.10	-0.10
95% CI	[-0.25 - 0.00]	[-0.28 - -0.03]*	[-0.24 - 0.01]	[-0.23 - 0.02]	[-0.23 - 0.03]
Mexican	-0.09	-0.10	0.01	0.05	0.03
95% CI	[-0.09 - -0.08]**	[-0.10 - -0.10]**	[0.01 - 0.01]**	[0.04 - 0.05]**	[0.03 - 0.04]**
Cuban	-0.03	-0.02	0.05	0.03	0.02
95% CI	[-0.04 - -0.02]**	[-0.03 - -0.01]**	[0.04 - 0.06]**	[0.02 - 0.04]**	[0.01 - 0.03]**
American Indian	0.02	0.01	0.01	0.03	0.08
95% CI	[0.01 - 0.03]**	[0.00 - 0.02]**	[-0.00 - 0.02]	[0.02 - 0.04]**	[0.07 - 0.09]**
Samoan	0.31	0.28	0.06	-0.04	-0.03
95% CI	[0.28 - 0.35]**	[0.24 - 0.31]**	[0.02 - 0.09]**	[-0.07 - -0.00]*	[-0.07 - 0.00]

BMI: body mass index, 95% CI: 95% confidence interval * $P < 0.05$; ** $P < 0.01$
